# Sensitivity and Specificity of the ECAS in Parkinson's Disease and Progressive Supranuclear Palsy

**DOI:** 10.1155/2018/2426012

**Published:** 2018-05-09

**Authors:** Jennifer A. Foley, Elaine H. Niven, Andrew Paget, Kailash P. Bhatia, Simon F. Farmer, Paul R. Jarman, Patricia Limousin, Thomas T. Warner, Huw R. Morris, Thomas H. Bak, Sharon Abrahams, Lisa Cipolotti

**Affiliations:** ^1^National Hospital for Neurology and Neurosurgery, Queen Square, London, UK; ^2^UCL Institute of Neurology, Queen Square, London, UK; ^3^School of Social Sciences (Psychology), University of Dundee, Dundee, UK; ^4^Reta Lila Weston Institute of Neurological Studies, UCL Institute of Neurology, Queen Square, London, UK; ^5^Department of Clinical Neuroscience, UCL Institute of Neurology, Queen Square, London, UK; ^6^Human Cognitive Neuroscience–PPLS, University of Edinburgh, Edinburgh, UK; ^7^Centre for Cognitive Ageing and Cognitive Epidemiology, University of Edinburgh, Edinburgh, UK; ^8^Anne Rowling Regenerative Neurology Clinic, University of Edinburgh, Edinburgh, UK; ^9^Dipartimento di Scienze Psicologiche, Pedagogiche e della Formazione, Università degli Studi di Palermo, Palermo, Italy

## Abstract

Disentangling Parkinson's disease (PD) and progressive supranuclear palsy (PSP) may be a diagnostic challenge. Cognitive signs may be useful, but existing screens are often insufficiently sensitive or unsuitable for assessing people with motor disorders. We investigated whether the newly developed ECAS, designed to be used with people with even severe motor disability, was sensitive to the cognitive impairment seen in PD and PSP and able to distinguish between these two disorders. Thirty patients with PD, 11 patients with PSP, and 40 healthy controls were assessed using the ECAS, as well as an extensive neuropsychological assessment. The ECAS detected cognitive impairment in 30% of the PD patients, all of whom fulfilled the diagnostic criteria for mild cognitive impairment. The ECAS was also able to detect cognitive impairment in PSP patients, with 81.8% of patients performing in the impaired range. The ECAS total score distinguished between the patients with PSP and healthy controls with high sensitivity (91.0) and specificity (86.8). Importantly, the ECAS was also able to distinguish between the two syndromes, with the measures of verbal fluency offering high sensitivity (82.0) and specificity (80.0). In sum, the ECAS is a quick, simple, and inexpensive test that can be used to support the differential diagnosis of PSP.

## 1. Introduction

It has now been over 50 years since progressive supranuclear palsy (PSP) was first described as a progressive neurological disorder with motor, ocular, and cognitive features [1]. Clinically, it remains difficult to distinguish from Parkinson's disease (PD) [2, 3], particularly in the early stages [4]. Even when using agreed criteria, the accuracy of diagnosis is not 100% [5]. As it has a significantly worse prognosis than PD, with a more rapid progression [6], early detection is crucial for enabling access to appropriate interventions and support, as well as identifying patients suitable for clinical trials. In the absence of any disease-specific biomarkers, there is a need for a quick, simple, and inexpensive test that can be used for the differential diagnosis of PSP.

Both PSP and PD are characterised by extrapyramidal syndromes, each of which can comprise symptoms of bradykinesia, rigidity, and/or postural instability [7]. Both disorders can feature eye movement abnormalities, and although the presence of the supranuclear vertical gaze palsy in PSP is diagnostically helpful, it is not universal [8, 9] and may be absent until quite late in the disease [10]. Although both disorders are thought to feature some similar cognitive signs, there is evidence to suggest that the specific cognitive profile may be a useful distinguishing feature [11].

Early cognitive impairment is a feature of PSP, which may precede the motor or ocular signs [12]. The profile is mainly that of executive dysfunction [13] and cognitive slowing [14], with markedly reduced verbal fluency [15, 16]. Deficits in other domains, including memory [17], language [18–20], visuospatial [16, 18], and social cognition [21–23], have also been reported.

In contrast, early stages of PD are characterised by only mild deficits in executive functions [24–27], but with illness progression, there is evolution from a mild cognitive impairment (MCI) to dementia, with greater involvement of posterior-based visual functions [28–30].

Existing screens of cognitive functioning can be criticised for being insufficiently sensitive to the cognitive profile of both PD and PSP. For example, the most widely used cognitive screen, the MMSE [31], has no measure of verbal fluency. Both the MMSE and the Addenbrooke's Cognitive Examination [32] have inadequate assessment of executive function. Their ensuing ceiling effects give them a low detection rate for cognitive impairment in Parkinsonian syndromes [33–35]. The Frontal Assessment Battery [36] does assess verbal fluency and executive function but has no measure of memory, language, or visuospatial function. Similarly, the Dementia Rating Scale (DRS) [37] has no measure of language or visuospatial function. This reliance upon the executive functions reduces its ability to discriminate PSP from PD [38] or frontotemporal syndromes [39]. The DRS also has a lengthy administration time and requires specialised testing materials, impractical for routine bedside use. The Montreal Cognitive Assessment [40] does have a measure of verbal fluency but does not accommodate for physical disability. Indeed, none of the existing assessments were designed specifically for people with movement disorders, such as Parkinsonian syndromes. Tasks involving speaking, writing, or drawing can be influenced by motor symptoms such as tremor, rigidity, bradykinesia, apraxia, or dysarthria; thus, a genuine cognitive impairment might be sometimes difficult to distinguish from motor dysfunction and performance decrements exaggerated by physical disability.

The ECAS [41] was recently developed as a brief assessment for the identification of cognitive and behavioural changes in disorders characterised by prominent motor symptoms, such as amyotrophic lateral sclerosis (ALS). It was developed to be used with patients with even severe physical disability and thus may be suitable for detecting cognitive impairment in all motor disorders. Many of the subtests can be performed either orally or manually, with some measures corrected for motor speed, reducing the impact that physical disability may have upon performance on cognitive tests [42]. It also allows the clinician to track cognitive impairment throughout the disease course, crucial for any longitudinal studies.

The ECAS has been standardised using a sample of healthy controls, providing normative data for clinical use [41]. It has also been validated against other screening tools [43, 44] and extensive neuropsychological assessment [45]. It is available in English [41], German [46], Swiss German [46], Italian [44], and Chinese [47]. However, it remains untested whether the ECAS is also sensitive to the cognitive impairment observed in other progressive movement disorders. Thus, the aims of the present study were to determine firstly whether the ECAS is sensitive to the cognitive impairment seen in PD and PSP and secondly whether it is able to distinguish between these disorders, in order to support the differential diagnosis of PSP.

## 2. Methods

### 2.1. Participants

All patients were recruited from the National Hospital for Neurology and Neurosurgery, Queen Square, London. PSP patients (9 males and 2 females) were diagnosed using the NINDS-SPSP criteria [48] and had a mean illness duration of 3.73 years (range 1–11 years). PD patients (24 males and 6 females) fulfilled the Queen Square Brain Bank criteria for PD and had a mean illness duration of 5.67 years (range 0–14 years). All patients with PD-MCI were identified using the Movement Disorder Society Task Force guidelines [49], in which impairment (<2 SD) is present on at least two tests of cognitive functioning, either within or across different cognitive domains.

The healthy controls were those reported by Niven et al. [45]. They (26 males and 14 females) were recruited through the Psychology Department of the University of Edinburgh. No participant had significant neurological or psychiatric history.

The research was done in accordance with the Declaration of Helsinki and approved by the NRES Committee London-Queen Square and the University of Edinburgh's Department of Psychology Ethics Committee.

### 2.2. Measures

The ECAS is a 15–20-minute screen that includes assessment of the following domains: (1) fluency (free: words beginning with “S” and fixed: words beginning with “T” but with only four letters); (2) executive functions, separate from verbal fluency (Reverse Digit Span, Alternation, Inhibitory Sentence Completion, and Social Cognition); (3) language (Naming, Comprehension, and Spelling); (4) memory (Immediate Recall, Delayed Percentage Retention, and Delayed Recognition); and (5) visuospatial (Dot Counting, Cube Counting, and Number Location). Verbal fluency measures take into account the slowing of motor responses, by generating a verbal fluency index corrected for motor speed. Previously published ECAS normative data [41] were used to classify the abnormality of performance on each domain and calculate the total score out of a maximum of 136 (lower score indicating worse performance), with any scores <2 SD considered to be impaired.

Extensive neuropsychological testing was administered to assess the same domains (fluency, executive functions, language, memory, and visuospatial; Table 1). Mood was assessed using the Hospital Anxiety and Depression Scale [50], and patients were also assessed using the Apathy Scale [51].

Scores for the neuropsychological assessments were compared with published normative data. For each measure, patients were judged to be impaired if scores were ≤2 SD. In the case where multiple measures were used, performance was classified as impaired when ≤2 SD on one of the two or two of the three measures was used.

### 2.3. Statistical Analyses

Data were analysed using SPSS v.19. Between-group comparisons were made using analyses of variance, and Pearson's and Spearman's correlations were used to detail the relationships between measures. Receiver operating characteristic (ROC) curve analyses were used to determine the relative sensitivity and specificity of the ECAS for the two patient groups.

## 3. Results

### 3.1. Demographics

Demographic details are given in Table 2. There were no significant group differences in age or education, and patients did not differ in symptom duration. There were significant group effects found for both HADS Anxiety (*F* (2, 75) = 13.04; *p* < 0.001) and Depression (*F* (2, 77) = 19.03; *p* < 0.001), with post hoc analysis revealing that patients had significantly higher burden of symptoms than healthy controls but no significant group difference between patient groups. There was no significant group difference in apathy scores between patient groups.

### 3.2. Performance on the ECAS

There was a significant effect of diagnosis on ECAS performance (Table 3). PSP patients had significantly lower total scores than PD patients and healthy controls, and PD patients had significantly lower total scores than healthy controls (all *p* < 0.017). There was a significant effect of diagnosis on all domains, except visuospatial. PSP patients performed worse than PD patients and healthy controls on fluency, language, executive function, and memory (all *p* < 0.017). PD patients performed worse than healthy controls on executive function only (*p* < 0.017).

When compared to published normative data, 81.8% (*n*=9) of the PSP patients and 30.0% of the PD patients (*n*=9) were impaired on the ECAS. PSP patients demonstrated most frequent impairments in fluency, language, and memory (each *n*=7; 63.6%) and then executive function (*n*=6; 54.5%) and visuospatial (*n*=3; 27.3%). PD patients demonstrated most frequent impairments in language (*n*=9; 30.0%), executive function and memory (each *n*=8; 26.7%), and then fluency and visuospatial (each *n*=5; 16.7%). There were no significant correlations between duration of symptoms and ECAS scores in either patient groups.

In order to investigate the specific nature of the impairment in both patient groups, individual domains were further investigated. In fluency, post hoc comparisons revealed a significant effect of diagnosis on both free fluency (*F* (2, 23.16) = 15.19; *p* < 0.017) and fixed fluency (*F* (2, 21.63) = 8.30; *p* < 0.017), with PSP patients performing worse than PD patients and healthy controls (all *p* < 0.017), but with no significant differences between PD patients and healthy controls. In language, there was a significant group effect on spelling (*F* (2, 76) = 10.58; *p* < 0.017), with PSP patients performing worse than PD patients and healthy controls (both *p* < 0.017), but with no significant difference between PD patients and healthy controls. In memory, there was a significant group effect on immediate recall (*F* (2, 24.04) = 11.47; *p* < 0.017) and retention (*F* (2, 20.69) = 8.92; *p* < 0.017), but not recognition. PSP patients performed significantly worse than PD patients and healthy controls on both of these (both *p* < 0.017), but with no significant difference between PD patients and healthy controls. In executive functions, there were significant group effects on reverse digit span (*F* (2, 25.96) = 7.60; *p* < 0.017), alternation (*F* (2, 22.92) = 5.66; *p* < 0.017), and social cognition (*F* (2, 20.42) = 9.49; *p* < 0.017). Both PSP and PD patients performed significantly worse than healthy controls on reverse digit span and social cognition (all *p* < 0.017), but with no significant differences between patient groups. PSP patients performed significantly worse than PD patients and healthy controls on alternation (*p* < 0.017), but with no significant difference between PD patients and healthy controls.

### 3.3. Performance on Full Neuropsychological Assessment

Upon full neuropsychological assessment, there was a significant effect of diagnosis on fluency, executive function, and visuospatial domains (Table 4). Specifically, there were significant group differences on both measures of fluency, with PSP patients performing worse than PD patients and healthy controls. There were no significant differences between PD patients and healthy controls. In addition, PSP patients performed worse than healthy controls on the Reading the Mind in the Eyes Test and Cube Analysis, but with no significant differences between PSP and PD patients, or between PD patients and healthy controls.

When scores on each of the neuropsychological assessments were compared with published normative data, there was a significant group difference in incidence of impairment in one domain only: fluency (*χ*^2^ (1) = 7.61; *p* < 0.001). Nine of the 11 PSP patients (81.8%) were classified as impaired on at least one measure of verbal fluency, in comparison with only 33.3% (*n*=10) of the 30 PD patients.

### 3.4. Diagnostic Accuracy of the ECAS for Cognitive Impairment in PD

Within the PD patients, a total of 17 (56.67%) met the criteria for PD-MCI. When PD and PD-MCI groups were compared, there were no significant differences in age, education, or symptom duration. However, on the ECAS, the PD-MCI group had significantly lower total scores (*t* (20.25) = 5.14; *p* < 0.001) and performed worse on executive function (*t* (23.83) = 3.02; *p* < 0.01), verbal fluency (*t* (22.52) = 3.26; *p* < 0.01), memory (*t* (28) = 3.09; *p* < 0.01), and visuospatial subscales (*t* (16.00) = 3.11; *p* < 0.01). On full neuropsychological assessment, the PD-MCI group also performed significantly worse on the Brixton Test (*t* (24) = 3.81; *p* < 0.001), Reading the Mind in the Eyes Test (*t* (26) = 2.75; *p* < 0.05), Graded Naming Test (*t* (28) = 4.32; *p* < 0.001), and Cube Analysis (*t* (20.69) = 2.801; *p* < 0.05).

ROC curve analysis revealed that the total score of the ECAS is able to discriminate between PD and PD-MCI with high sensitivity (88.2%) and 100% specificity, when using a threshold score of 112.50/136. The AUC is 0.93 (SE = 0.06; *p* < 0.001). Confidence intervals are 0.81 (lower bound) and 1.00 (upper bound). Indeed, all PD patients who performed in the impaired range on the ECAS fulfilled the diagnostic criteria for PD-MCI.

### 3.5. Diagnostic Accuracy of the ECAS for PSP

ROC curve analysis also revealed that the ECAS is highly specific (86.8%) and sensitive (91.0%) when discriminating PSP patients from healthy controls using a threshold score of 113.50/136. The AUC is 0.91 (SE = 0.67; *p* < 0.001). Confidence intervals are 0.79 (lower bound) and 1.00 (upper bound). All PSP patients who performed in the impaired range on the ECAS demonstrated impairment upon full neuropsychological testing, including impairment on at least one measure of verbal fluency.

### 3.6. Diagnostic Accuracy of the ECAS for Distinguishing between PD and PSP

**Figure 1:**
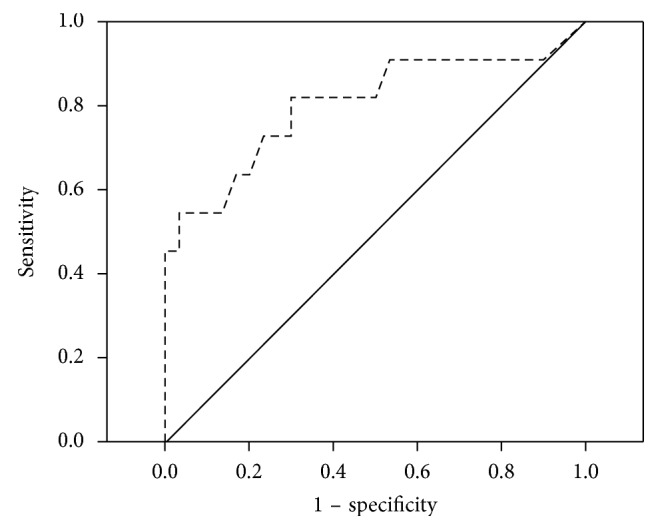
ROC curve depicting sensitivity and specificity of the ECAS, when comparing the PD and PSP patients (higher sensitivity scores indicate lower performance).

The second aim of the study was to determine whether the ECAS is able to distinguish between PD and PSP, in order to support the early and accurate diagnosis of PSP. ROC curve analysis showed that the measure is able to discriminate between PD and PSP (when comparing all patients, irrespective of cognitive performance), with high specificity (76.7%) and sensitivity (72.7%), using a threshold score of 103.50/136 (Figure 1). The AUC is 0.80 (SE = 0.09; *p* < 0.01). Confidence intervals are 0.62 (lower bound) and 0.98 (upper bound). This generated three false negatives and seven false positives. The false positives were all patients who fulfilled the criteria for PD-MCI.

Within the ECAS, fluency was the best predictor of PSP, with high specificity (80.0%) and sensitivity (82.0%) using a threshold score of 17/24. The AUC is 0.84 (SE = 0.08; *p* < 0.01). Confidence intervals are 0.69 (lower bound) and 1.00 (upper bound). This generated two false negatives and six false positives (five PD-MCI and one PD). This is in contrast to when using the raw number of words generated in the two fluency tasks as a predictor, which has lower sensitivity (77.8%) and specificity (79.2%) when using a threshold score of 17.5.

## 4. Discussion

Our study has shown that the ECAS is sensitive to the cognitive impairment seen in PD. We found that 30% of PD patients were impaired on the ECAS, all of whom also demonstrated impairments upon full neuropsychological testing and fulfilled the criteria for PD-MCI. Indeed, ROC curve analyses revealed that the ECAS has excellent sensitivity and complete specificity for detection of PD-MCI. On the ECAS, PD patients demonstrated impairments in a number of domains but performed significantly worse than healthy controls on one domain only: executive function. PD-MCI patients also demonstrated deficits in language and visuospatial functioning. These findings confirm the greater involvement of posterior functions with more advanced Parkinson's disease [30] but also suggest that the pattern of impairment can be fairly heterogeneous, even involving language. This is in accordance with the findings of the MDS Task Force [49], who also report impairments in a range of cognitive domains, including language.

Our data also show that the ECAS is sensitive to the cognitive impairment in PSP. We found that 81.8% of PSP patients were impaired on both the ECAS and full neuropsychological testing, including at least one measure of fluency. Again, ROC curve analyses confirmed that the ECAS total score gave excellent sensitivity and specificity for detection of PSP when compared with healthy controls. On the ECAS, PSP patients demonstrated the expected impairment in fluency, but also executive function, memory, and language. On extensive neuropsychological testing, PSP patients demonstrated impairments in fluency as well as executive function and visuospatial. The prominence of fluency and executive impairment on both the ECAS and full neuropsychological testing is in accordance with previous descriptions [13, 15, 16, 28], confirming that the ECAS is sensitive to the typical profile of cognitive impairment in PSP.

Importantly, we also found that the ECAS was able to distinguish between PD and PSP. ROC curve analysis revealed that the ECAS total score was sensitive and specific to PSP, with verbal fluency being the best discriminator. The ECAS was able to identify all PSP cases demonstrating cognitive impairment upon full testing. The few false positives mostly reflected PD patients with advanced cognitive impairment.

The strikingly reduced verbal fluency found on the ECAS and full testing confirms this as the cognitive hallmark of PSP. Importantly, the ECAS revealed this marked deficit even after accounting for the slowed motor speed. This contrasts with impairments in other cognitive domains, such as memory, which can improve by up to 50% given sufficient extra time [58, 59]. This impoverished verbal fluency, alongside the frequent family reports of reduced spontaneous speech and conversation initiation, likely reflects a cognitive adynamia beyond that of simple bradyphrenia but rather a more significant impairment in the generation of a “fluent sequence of novel thought” [19, 60]. This may reflect a deficit in novel thought generation and/or its appropriate sequencing [61]. Indeed, it has been argued that the akinesia in motor abilities, reduction of verbal fluency in cognition, and apathy in behaviour are all different manifestations of the same underlying disorder [62].

PSP patients also demonstrated impairments in other domains, which supports the findings of previous studies. In accordance with previous reports of poor delayed recall [17], our PSP patients displayed impaired verbal recall on the ECAS, with three of the seven PSP patients impaired on both the ECAS and full testing. Our patients also demonstrated language impairment, reflecting spelling difficulties, in accordance with previous studies [20, 63, 64]. Spelling was more impaired on the ECAS, perhaps because its spelling test comprises nouns, verbs, and compounds of low-to-medium frequency, with a longer mean length. In contrast, the spelling test used upon full testing contained mostly nouns of high-to-low frequency, with a shorter mean length. Our patients also demonstrated impaired visuospatial function, which supports previous findings [16, 18]. Nearly a third of PSP patients were impaired on the ECAS, with all of these also impaired upon full testing.

The PD and PSP patients both performed poorly on measures of social cognition. These findings echo previous reports of impaired performance on tests of theory of mind and social norms [22, 44, 65–67].

## 5. Conclusions

The ECAS captures the core cognitive deficit of reduced verbal fluency, as well as the wider cognitive profile of PSP. This may allow longitudinal testing to track progression as verbal fluency reaches floor. It was possible to use the ECAS with all the patients who took part in this study, despite often severe motor symptoms, indicating that it would be well tolerated in those with advanced disease. This suggests that the ECAS is suited for bedside use for detecting cognitive impairment in Parkinsonian syndromes and for distinguishing different cognitive profiles within these, in order to support differential diagnosis. Full neuropsychological assessment can then be used to further elucidate the specific clinical profile of each patient. Future research should examine its sensitivity for detecting cognitive impairment in other progressive movement disorders.

## Figures and Tables

**Table 1:** Neuropsychological assessment.

Domain	Subdomain	Measures
Fluency		Phonemic verbal fluency index [42] (VFi): words beginning with “P” and “R”

Executive functions	Inhibition	Hayling Sentence Completion Test [52]: total unconnected errors (converted but not scaled); latency score (time taken to complete unconnected sentences minus time taken for connected sentences)
	Shifting and rule detection	Brixton Spatial Anticipation Test [52]: total number of errors
	Social	Reading the Mind in the Eyes–Revised [53]: total number of correct ones

Language	Naming	Graded Naming Test [54]
	Spelling	Graded Difficulty Spelling Test [55]

Memory		Adult Memory and Information Processing Battery [56]: immediate story recall; delayed story recall

Visuospatial		The Visual Object and Space Perception Battery [57]: cube analysis; number location

**Table 2:** Demographics of the participants.

	PSP patients		PD patients		Controls	
	Mean (SD)	Range	Mean (SD)	Range	Mean (SD)	Range
Age	66.82 (7.08)	53–77	63.33 (7.89)	50–80	62.70 (10.48)	39–88
Duration of symptoms (years)	3.73 (3.20)	1–11	5.67 (3.47)	0–14	—	—
Gender (female)	2	—	6	—	14	—
Education, mean years (SD)	15.27 (4.98)	10–26	14.33 (3.22)	9–23	12.25 (3.39)	9–25
HADS Depression	8.50 (5.50)	1–15	6.77 (4.39)	0–16	2.40 (1.81)	0–6
HADS Anxiety	8.50 (5.79)	2–17	9.17 (4.13)	3–18	4.83 (2.75)	0–11
Apathy	17.67 (12.04)	4–34	15.90 (10.26)	4–42	—	—

HADS: Hospital Anxiety and Depression Scale.

**Table 3:** ECAS scores of the participants.

	PSP patients		PD patients		Controls		*F* (df)	*p*
	Mean (SD)	Range	Mean (SD)	Range	Mean (SD)	Range		
Total (max. 136)	85.09 (24.46)	54–126	109.87 (13.52)	78–126	120.61 (7.06)	100–132	17.44 (2, 22.09)^a^	<0.001
Executive function (max. 48)	29.27 (13.27)	9–46	36.93 (6.05)	19–44	42.11 (3.49)	33–48	12.56 (2, 22.22)^a^	<0.001
Language (max. 28)	23.18 (5.06)	15–28	26.67 (2.11)	19–28	27.50 (0.80)	25–28	5.76 (2, 21.01)^a^	<0.001
Fluency (max. 24)	8.18 (8.74)	0–22	18.73 (4.83)	6–24	20.74 (2.39)	12–24	12.37 (2, 21.91)^a^	<0.001
Memory (max. 24)	10.27 (6.90)	0–20	15.67 (4.25)	3–22	18.39 (2.53)	13–23	10.85 (2, 22.76)^a^	<0.001
Visuospatial (max. 12)	11.09 (1.87)	6–12	11.47 (1.04)	8–12	11.87 (0.67)	8–12	2.33 (2, 22.86)^a^	0.066

^a^Welch's adjusted *F* ratio.

**Table 4:** Neuropsychological assessment performance of participants.

		PSP patients		PD patients		Healthy controls		*F* (df)	*p*	Post hoc
		Mean (SD)	Range	Mean (SD)	Range	Mean (SD)	Range			
Fluency	“P” VFi	10.90 (6.30)	2.37–19.67	4.13 (2.98)	0.96–14.50	3.68 (1.96)	1.52–9.33	22.65 (2.78)	<0.001	PSP < PD
	“R” VFi	14.16 (13.16)	2.50–41.00	4.12 (2.11)	2.00–9.50	3.65 (1.73)	1.72–9.33	22.37 (2.76)	<0.001	PSP < PD

Executive function	Hayling: B–A time	69.14 (86.55)	5–251	33.00 (28.04)	−3 to 126	34.88 (28.96)	−5 to 121	2.88 (2.73)	0.06	
	Hayling: errors	6.57 (5.56)	0–14	5.17 (5.89)	0–29	8.75 (9.17)	0–32	1.80 (2.73)	0.17	
	Brixton	38.43 (3.21)	35–43	34.54 (9.71)	16–50	35.08 (8.23)	15–47	0.59 (2.70)	0.56	
	Reading the Mind in the Eyes	20.50 (5.43)	14–29	23.93 (5.06)	13–35	26.35 (3.81)	17–34	5.72 (2.71)	<0.01	PSP < HC

Language	Graded Naming Test	20.64 (5.85)	9–26	23.20 (3.61)	13–29	24.15 (6.64)	14–57	1.71 (2.78)	0.19	
	Graded Difficulty Spelling Test	19.64 (8.44)	2–29	22.29 (6.01)	7–30	22.53 (4.50)	12–29	1.15 (2.76)	0.32	

Memory	Immediate Story Recall	24.45 (14.20)	0–41	27.04 (9.73)	7–49					
	Delayed Story Recall	23.64 (15.54)	0–40	25.46 (9.61)	7–46					
	Retention	80.16 (32.96)	0–111.11	93.92 (14.75)	62.50–136.00	94.49 (12.74)	58.82–12.27	3.12 (2.77)	0.05	

Visuospatial	Cube Analysis	8.91 (1.58)	6–10	8.83 (1.62)	5–10	9.63 (0.87)	5–10	23.61 (2.78)	<0.05	PSP < HC
	Number Location	8.45 (2.46)	2–10	9.20 (1.00)	7–10	9.43 (0.71)	7–10	2.90 (2.78)	0.06	

VFi: Verbal Fluency Index.
